# Taxonomic validation of five fish species of subfamily Barbinae from the Ganga river system of northern India using traditional and truss analyses

**DOI:** 10.1371/journal.pone.0206031

**Published:** 2018-10-26

**Authors:** Deepmala Gupta, Arvind Kumar Dwivedi, Madhu Tripathi

**Affiliations:** 1 Department of Zoology, University of Lucknow, Uttar Pradesh, India; 2 Wildlife Institute of India, Dehradun, Uttarakhand, India; Florida State University, UNITED STATES

## Abstract

Morphometric differences were investigated among five fish species of subfamily Barbinae from the Ganga river system through traditional morphometrics and the truss network system. Species taken into account were *Puntius chola* (Hamilton 1822), *Puntius sophore* (Hamilton 1822), *Pethia ticto* (Hamilton 1822), *Pethia conchonius* (Hamilton 1822) and *Systomus sarana* (Hamilton 1822). Although, taxonomists carefully examine external body features to discriminate these species, there is still a risk of misidentification during a visual assessment. In the present study, the traditional morphological analysis included 22 morphometric measurements and 10 meristic counts. Truss network system of 14 landmarks was interconnected to yield 91 distance variables. The principal component analysis (PCA), discriminant function analysis (DFA) and cluster analysis (CA) were employed in order to determine morphometric variations. In traditional analysis, 29 characters out of 32 were found significant (*p*<0.05). Eight principal components were extracted through PCA explaining 85.30% of the total variance in samples, DFA correctly classified 100.0% of original grouped cases and 100.0% of cross-validated grouped cases. Truss analysis showed that all the 90 characters were significant (*p*<0.05). PCA extracted four principal components explaining 96.45% of the total variance. DFA correctly classified 96.1% of original grouped cases and 92.1% of cross-validated grouped cases. The results acquired from the traditional as well as truss analyses indicate significant morphometric heterogeneity. However, variations are not the same for the two different methods (traditional and truss) employed for the analyses. Shape differences among species were evident from relative warps (RW) supporting truss network analysis. Geometric morphometric methods (GMM), but limited use of Procrustes methods revealed even very small dissimilarity between groups. In spite of determining the morphometric differentiation among species, the present study also provides a useful insight on the application and complementary role of truss analysis with traditional morphometric analysis in the correct classification of the selected species.

## Introduction

Barbinae is a taxonomic subfamily, within the family Cyprinidae, that belongs to order Cypriniformes. The Barbin fishes of genus *Puntius* are native to South Asia, Mainland Southeast Asia, and Taiwan [1]. *Puntius* has been familiar as a “catch-all” genus and encompasses more than 60 species found in India and new species are continuously being discovered [2–5]. The *Puntius* is an economically important ornamental, as well as a food fish locally sold fresh in markets. It is highly valued in recreational fisheries and constitutes a major component of the tropical fish trade. *Puntius sarana* (now allocated to *Systomus*) also plays a significant economic role in aquaculture [6–8]. In spite of its economic significance, comprehensive knowledge on systematic/taxonomy is still incomplete for this genus. Further, generic placement/status of particular species of *Puntius* genus remains questionable.

Many closely related *Puntius* species exhibit taxonomic indistinctness [9–10]. Therefore, interrelationships of this genus are not well inferred [2, 11–14]. Since the beginning, genus *Puntius* has undergone many serious nomenclature and systematic changes. Morphological characters have long been used to study the diversity and taxonomy of these cyprinids [15–16]. Unfortunately, taxonomic ambiguity in *Puntius* is yet to be resolved though several studies on systematic relationships have been done within the subfamily Barbinae [17–21]. Based on morphological characteristics, Rainboth [22, 23] classified *Puntius* into another three genera, *Systomus*, *Barbodes*, and *Hypsibarbus*. However, in contrast to Rainboth [22, 23], Champasri et al [24, 25] and Rajasekaran and Sivakumar [26] stated that *Puntius* should not be split into three genera as Rainboth [22, 23] failed to provide distinct special characters to differentiate three new genera within the *Puntius* genus. The division of *Puntius* into three genera by Rainboth [22, 23] has remained as a controversial issue among fish taxonomists. Later, on the basis of osteological studies, Shantakumar and Vishwanath [27] have documented new separate groups within the genus *Puntius*. Furthermore, Pethiyagoda et al [28] reported five well-supported clades as distinct genera within South Asian *Puntius* namely *Pethia*, *Dawkinsia*, *Dravidia*, *Systomus* and *Puntius*. Their study was based on two mitochondrial DNA gene fragments, external-morphological, and osteological characters. On the basis of their study Pethiyagoda et al [28] split the *Puntius* genus and allocated *Puntius sarana* to *Systomas*, *Puntius conchonious* and *Puntius ticto* to *Pethia*. Many other species (*P*. *barbodes*, *P*. *desmopuntius*, *P*. *haludaria*, *P*. *oliotius*, *P*. *puntigrus and P*. *sahyadria*) which were primarily placed within the *Puntius* genus have also been shifted to other genera [29–31].

Afterwards, Saroniya et al [32] studied the meristic characters of some of *Puntius* species of central India and revealed deviations from the earlier studies of different workers. Saroniya et al [33] on the basis of 18S rDNA sequences, disagree over the polyphyletic origin of genus *Puntius* and revealed closeness among *Puntius chola*, *Puntius sophore*, *Puntius ticto* and *Puntius conchonius*. Furthermore, as per IUCN Red List 2017 ver. 3.1, the generic status of *Puntius sarana* is still uncertain and continually shifting between *Barbodes* and *Puntius*. Although FishBase considered *Pethia ticto*, *Pethia conchonius*, *Systomus sarana* ‘valid’, still investigators are continuing with previously valid names [34–36].

Taxonomic tribulations within the *Puntius* group have been confirmed by many previously reported studies [37]. Morphometric characters have been efficiently employed for taxonomic related problems [38, 39]. The traditional morphometric method involves direct quantification of a range of morphological characters that are subsequently analyzed via multivariate methods [40–41]. This method is remarkably useful in identification of closely related species and provides preliminary insights on fish taxonomy [42]. Meristic characters were also found to be valid in the race and species identification for adequate aqua-management and fisheries statistics [43–45]. The traditional morphometric methods are coupled with some limitations in describing the fish shape, therefore this has been criticized [46, 47]. Consequently, a strong landmark-supported tool based on statistical analysis called ‘truss network technique’ is used for distinguishing species and this technique was extensively used by many workers in discrimination of species [48–53]. Geometric morphometrics analysis of landmarks from digital images is highly effective in capturing information about the shape of an organism [54, 55].

In this context, the present study aims to examine the morphometric variations among fishes of subfamily Barbinae available in the Ganga river system of northern India. The examined species are *Puntius chola* (Hamilton 1822) known as ‘swamp barb’, *Puntius sophore* (Hamilton 1822), known as ‘pool barb’, *Systomus sarana* (Hamilton 1822) known as ‘olive barb’, *Pethia ticto* (Hamilton 1822) known as ‘two spot barb’ and *Pethia conchonius* (Hamilton 1822), known as ‘rosy barb’. The study is based on traditional and truss network analyses with the objective to employ useful insights into morphometric characters responsible for morphometric and shape variations among the five selected fish species.

## Materials and methods

### Sample collection

**Fig 1:**
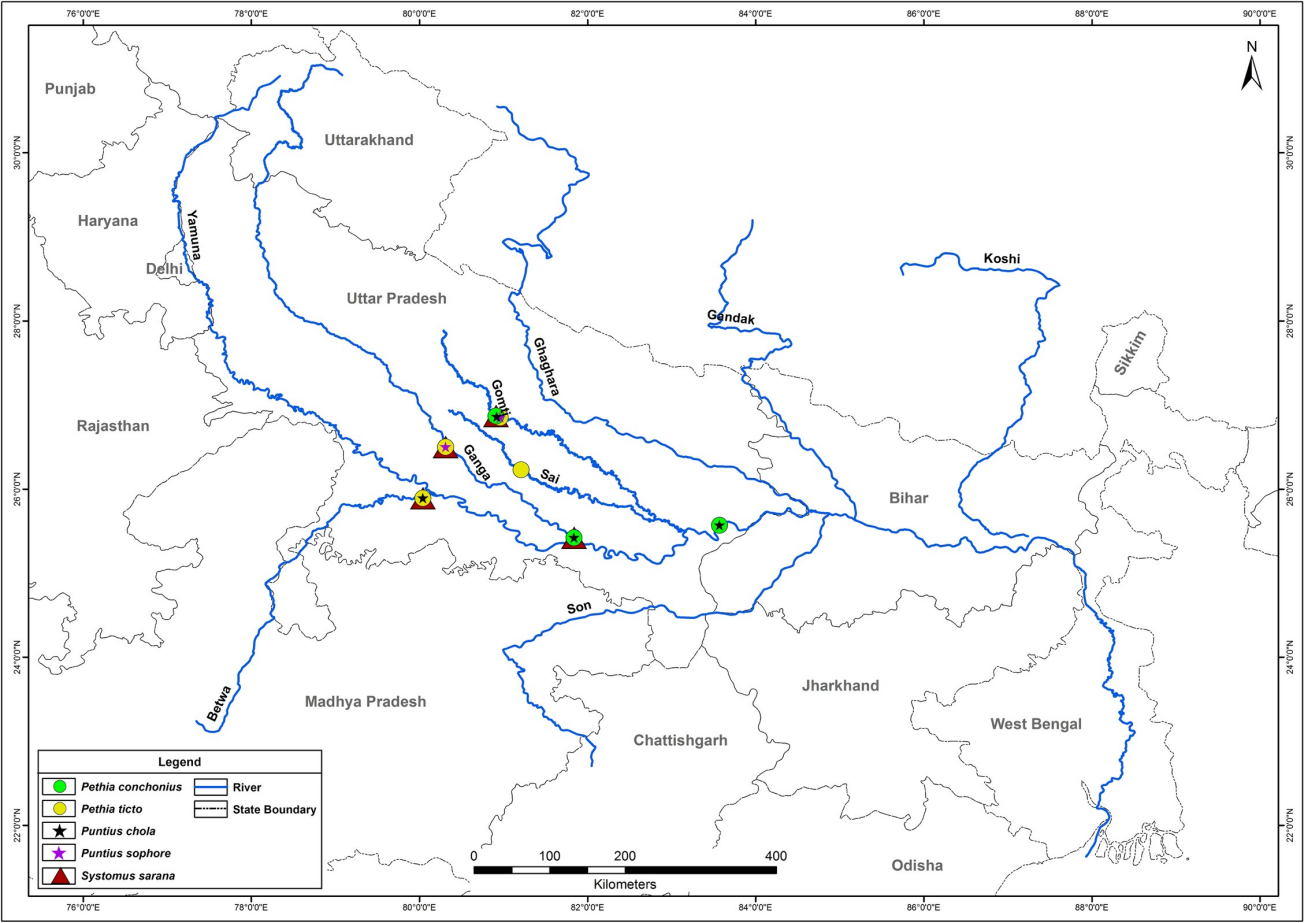
Map showing different sampling sites of selected species.

**Fig 2:**
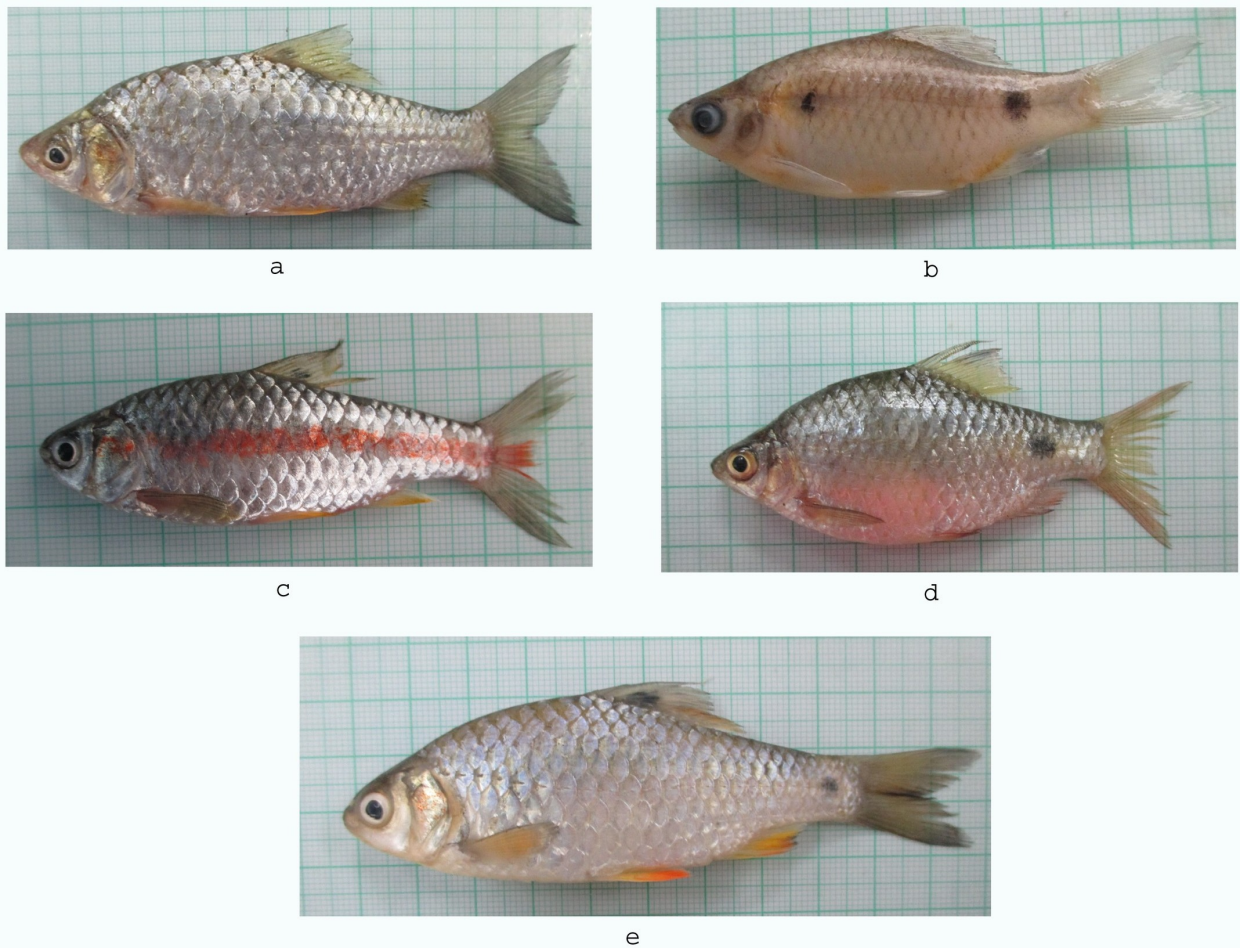
**Representative specimens of each species** (a) *S*. *sarana* (b) *P*. *ticto* (c) *P*. *sophore*, (d) *P*. *conchonius* and (e) *P*. *chola*.

Ninety fish samples of five species were collected from different fishing sites of Ganga river system in northern India (Fig 1), with the assistance of local fishermen during August, 2016 to December, 2016. Information about the number of samples collected, sampling sites, and geographical coordinates of locations for Barbinae species studied in the present study is provided in Table 1. Each fish specimen was immediately preserved in a 10% formalin solution and subsequently subjected for identification according to Talwar and Jhingran [56], Jayaram [57], Pethiyagoda and Kottelat [58]. Representative photographs of each species are showed in Fig 2.

**Table 1:** Sampling sites, their locations with latitude and longitude, sample size and size range of five species of Barbinae used in the present study.

Species	Sample size		River	Sampling sites	Latitude and Longitude	Standard Length (SL) range (cm)	Mean SL (cm)±SD (CV)	Mean Total Weight (TW) (g) ±SD
	Traditional morphometrics	Truss						
*Systomus sarana*	24	18	Ganga	Ganga barrage, Kanpur	26.50°N, 80.31°E	10.4–23.7	16.82 ±3.04 (18.10)	21.2 ±3.21
			Gomti	Pakka pul, Lucknow	26.87°N, 80.91°E			
			Betwa	Sahurapur daria, Hamirpur	25.89°N, 80.04°E			
			Yamuna	Mutthi ganj, Allahabad	25.42°N, 81.84°E			
*Pethia ticto*	20	20	Ganga	Ganga barrage, Kanpur	26.50°N, 80.31°E	4.8–6.0	5.45 ±0.32 (5.91)	4.56 ±1.78
			Gomti	Gomti barrage, Lucknow	26.85°N, 80.96°E			
			Sai	Takia kalan, Raebareli	26.23°N, 81.21°E			
			Betwa	Sahurapur daria, Hamirpur	25.89°N, 80.04°E			
*Pethia conchonius*	08	11	Yamuna	Mutthi ganj, Allahabad	25.42°N, 81.84°E	5.0–6.2	5.75 ± 0.45 (7.83)	5.54 ±2.89
			Ganga	Dadri Ghat, Gazipur	25.57°N, 83.57°E			
			Gomti	Pipe wala pul, Lucknow	26.87°N, 80.91°E			
*Puntius sophore*	06	20	Gomti	Gomti barrage, Lucknow	26.85°N, 80.96°E	6.0–7.8	7.13 ± 0.76 (10.63)	11.5 ±3.02
			Ganga	Ganga barrage Kanpur	26.50°N, 80.31°E			
			Betwa	Sahurapur daria, Hamirpur	25.89°N, 80.04°E			
*Puntius chola*	12	09	Ganga	Dadri Ghat, Gazipur	25.57°N, 83.57°E	7.6–8.6	8.18 ± 0.32 (3.96)	17.2 ±3.21
			Gomti	Saheed smarak, Lucknow	26.86°N, 80.92°E			
			Betwa	Sahurapur daria, Hamirpur	25.89°N, 80.04°E			
			Yamuna	Mutthi ganj, Allahabad	25.42°N, 81.84°E			

### Traditional morphometrics

#### Data collection

**Fig 3:**
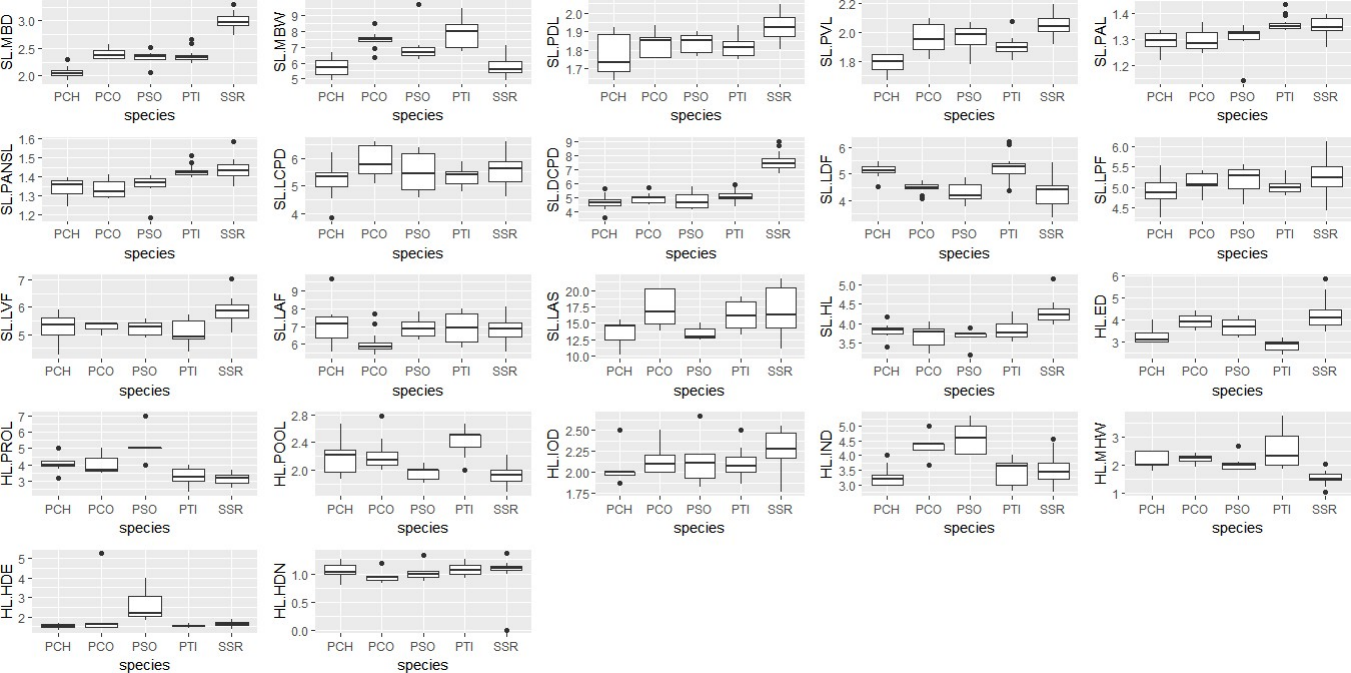
Boxplots of selected species based on morphometric characters: PCH (*P*. *chola*), PCO (*P*. *conchonius*), PSO (*P*. *sophore*), PTI (*P*. *ticto*), SSR (*S*. *sarana*) {SL: Standard length; MBD: Maximum body depth; MBW: Maximum body width; PDL: Pre-dorsal length; PVL: Pre-ventral length; PAL: Pre-anal length; PANSL: Pre-anus length; LCPD: Length of caudal peduncle; DCPD: Depth of caudal peduncle; LDF: Length of dorsal fin; LPF: Length of pectoral fin; LVF: Length of ventral fin; LAF: Length of anal fin; LPA: Length of pelvic axial; HL: Head length; ED: Eye diameter; PROL: Pre-orbital length; POOL: Post-orbital length; IOD: Inter-orbital distance; IND: Inter-narial distance; MHW: Maximum head width; HDE: Head depth at eye; HDN: Head depth at nape}. Fourteen characters were divided by SL, eight by HL, SL not shown.

In the laboratory, a total of 23 morphometric measurements (S1 Table) were recorded for each specimen (Fig 3). Measurements were made with vernier caliper closest to 0.1 mm. Counts and measurements were taken as far as possible on the left side of fish specimens following standard methods for cyprinid taxonomy [58] with some modifications.

#### Meristic characteristics

Ten meristic characteristics were counted. A comparison of meristic characters of five species is showed in Table 2. Meristic characters were counted twice by the same observer. Radiographs were taken using digital X-ray machine. The total number of vertebrae was counted from the radiographic images. The initial four fused vertebrae (weberian apparatus) were not incorporated in the vertebral counts.

**Table 2:** Meristic characters of five selected species of Barbinae with code, mean±SD and their significance level.

S. No.	Meristic character	Code	*S*. *sarana*	*P*. *sophore*	*P*. *chola*	*P*. *ticto*	*P*. *conchonius*	P-values
1	Scales in Lateral Series	SLL	31.708±1.083	24.000±0.894	26.417±1.379	24.200±0.894	25.125±0.991	0.000*
2	Scales in Transverse Series	STR	10.208±0.388	8.000±0.000	9.167±0.389	8.700±0.571	9.250±0.463	0.000*
3	Pre-dorsal Scales	PDS	10.958±0.204	8.667±0.516	9.083±0.793	10.500±0.513	8.875±0.641	0.000*
4	Caudal Circumferential Scales	CCS	14.971±1.018	12.000±0.632	12.167±0.835	11.150±0.366	11.875±0.354	0.000*
5	Dorsal Fin Rays	DFR	12.208±0.451	9.000±0.000	10.000±0.000	9.000±0.000	11.000±0.000	0.000*
6	Pectoral Fin Rays	PFR	14.250±0.532	15.000±0.000	14.833±0.389	11.700±0.470	12.625±0.744	0.000*
7	Pelvic Fin Rays	PFR	9.000±0.000	8.000±0.000	7.333±0.492	9.00±0.000	7.750±0.463	0.000*
8	Anal Fin Rays	AFR	9.125±0.448	9.000±0.000	9.000±0.000	6.000±0.000	9.000±0.000	0.040*
9	Caudal Fin Rays	CFR	19.208±0.415	19.000±0.000	19.000±0.000	19.000±0.000	19.000±0.000	0.032*
10	Vertebral column count	VCC	32.333±0.637	26.000±0.000	27.583±0.793	25.450±0.887	25.000±0.000	0.000*

*Significant at *p*<0.05

(P-values were calculated using SPSS)

### Landmark-based morphometrics

#### Data collection

**Fig 4:**
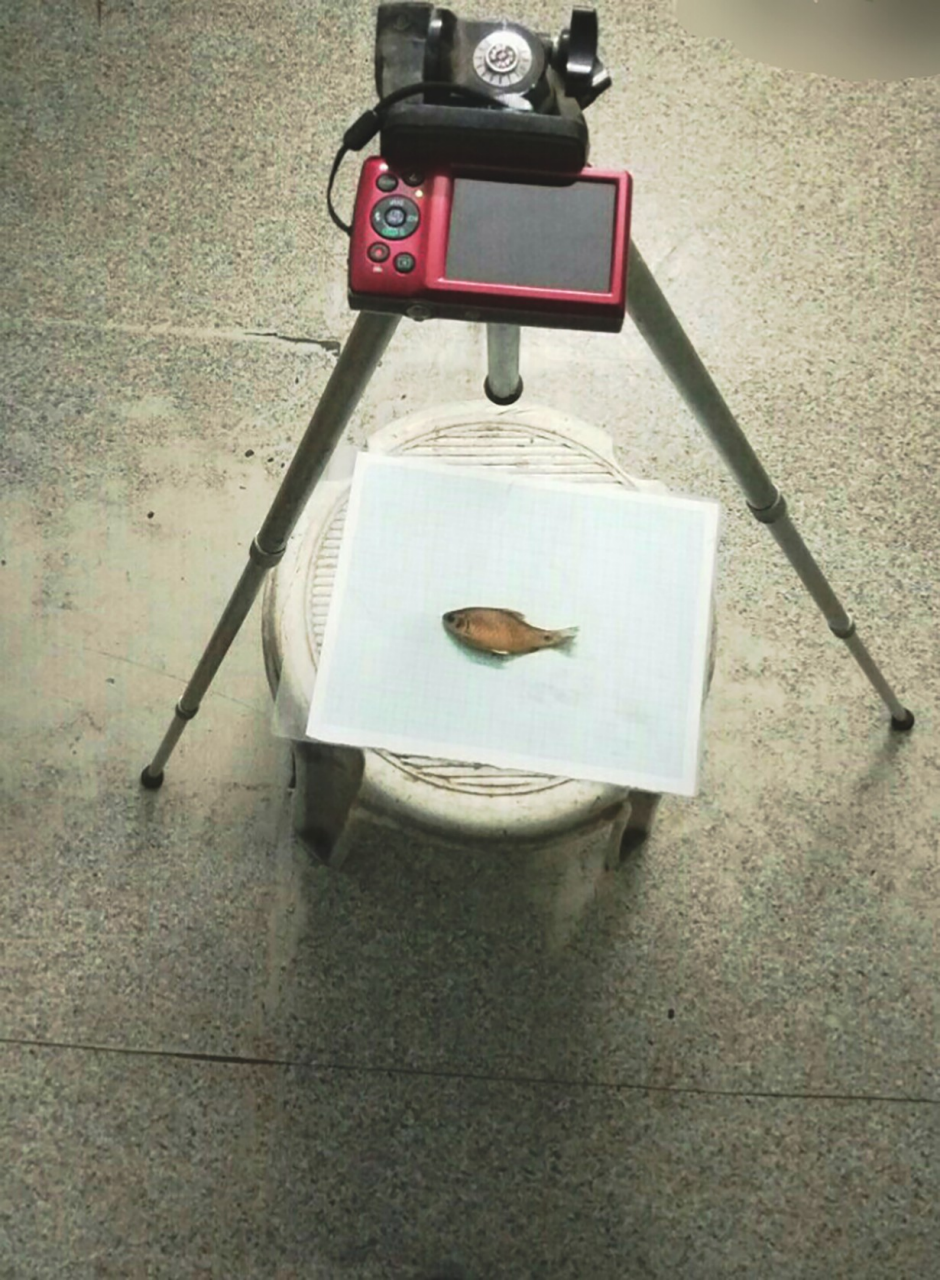
Imaging sample fish on laminated truss sheet for truss morphometrics.

Sampled specimens were placed on a flat surface on a plastic-coated graph paper, which was used for standardizing the coordinates of the digital images. Each fish sample was given a specific code for identification. A digital camera (Canon IXUS 145) was used to capture the digital images. All fish specimens were positioned laterally on their right side, with their body posture and fins teased into a natural position (Fig 4). Images of fish specimens were captured and were transferred to the computer for further analysis.

### Traditional morphometric analysis

Each of fourteen morphometric characters was divided by standard length (SL) and remaining 8 characters were divided by head length (HL) to eliminate the size effect (correlation < 0.5 for all variables). All the morphometric values were log-transformed prior to analysis using computer software PAST 1.47 (PAleontological Statistics) [59]. Multivariate statistical techniques, ANOVA, principal component analysis (PCA), discriminate function analysis (DFA) and cluster analysis (CA) were performed on log_10_-transformed measurements. PCA is an effective method for morphometric data reduction and extracting independent variables. DFA is a predictive model for group membership. The source for the discrimination among samples was based on the percentage of correctly and incorrectly classified fish. Eigenvalues, a percentage of variance, cumulative percentage and canonical correlation were acquired using correlation matrix from PCA. Statistical analyses were performed with the computer software programs SPSS 16.0 and PAST 1.47. A hierarchical cluster analysis based on UPGMA (Unweighted Pair Group Method with Arithmetic mean) was carried out on Mahalanobis distances. Software STATISTICA was used to test the significance of Mahalanobis distance between species.

### Truss-based morphometric analysis

**Fig 5:**
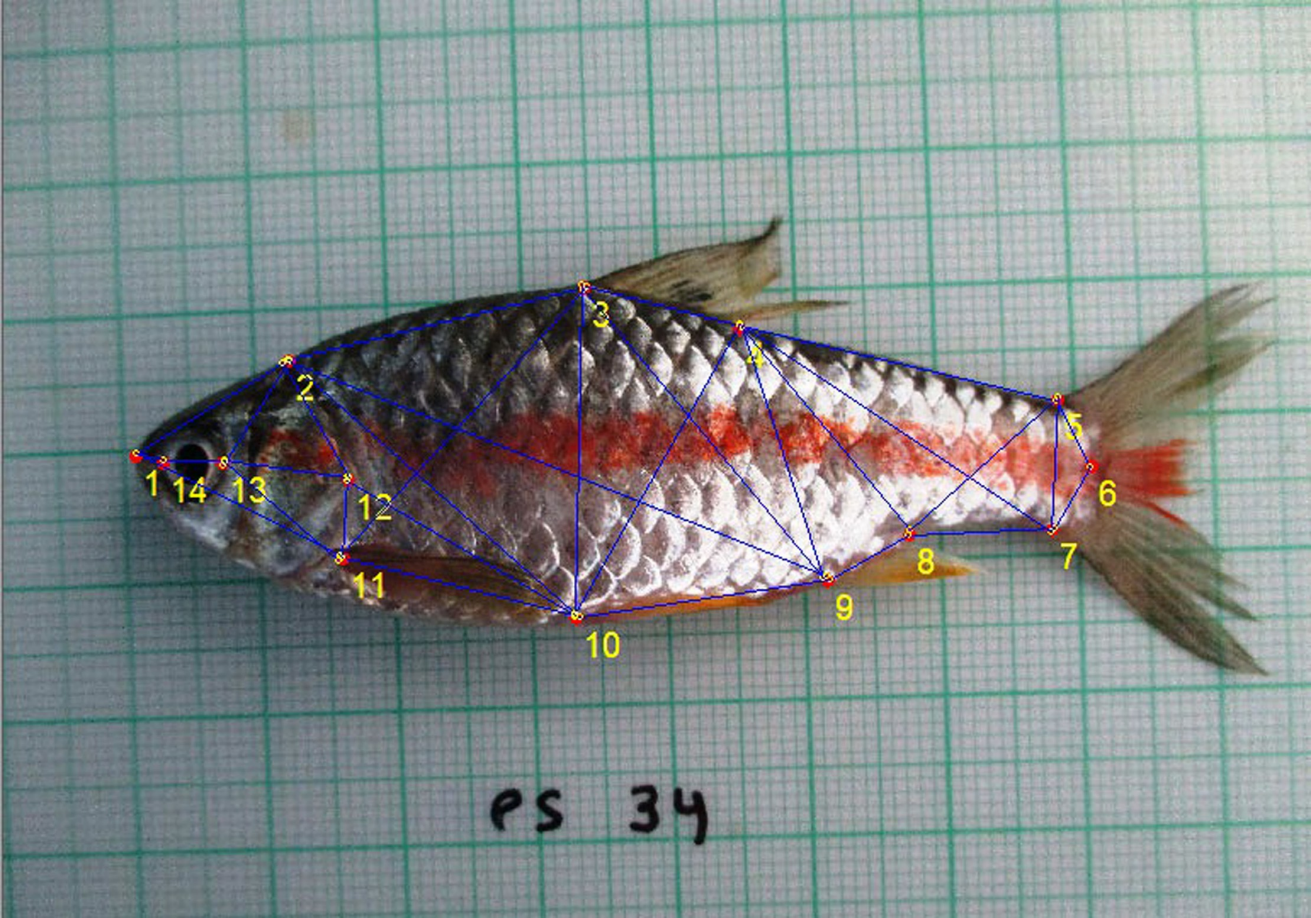
*Puntius sophore* representing locations of 14 landmarks and the distances measured which were used for morphological variations. Landmarks refer to: (1) anterior tip of snout at upper jaw (2) most posterior aspect of neurocranium (beginning of scaled nape) (3) origin of dorsal fin (4) end of dorsal fin (5) anterior attachment of dorsal membrane from caudal fin (6) posterior end of vertebrae column (7) anterior attachment of ventral membrane from caudal fin (8) end of anal fin (9) origin of anal fin (10) insertion of pelvic fin (11) insertion of pectoral fin (12) end of operculum (13) posterior end of eye (14) anterior end of eye.

The extraction of the truss distances from the digital images of specimens was done using a combination of the software platforms, tpsUtil, tpsDig 2 v2.1 and PAST [59–61]. Software tpsUtil converts JPEG image into tps format. For covering entire shape of fish specimen, two-dimensional Cartesian coordinates of 14 landmarks were recorded on the lateral view of each specimen (Fig 5). The locations of the landmarks were selected according to the following two criteria: reliability in terms of correspondence between specimens, and the ability to best describe the geometry of the form under study. All the landmarks were digitized and truss networks were constructed by interconnecting the landmarks using software tpsDig. Using the computerized Pythagorean theorem in software PAST, X–Y coordinate data was transformed into linear distances for subsequent analysis. Altogether, 91 morphometric characters were attained connecting these landmarks [60]. The truss data generated by PAST were log-transformed to conserve allometries and to standardize variances [62].

To eliminate size effect data were M-transformed by employing formula given below [63].

M‑trans=logM‑b(logSL‑logSLmean)

Where, M-trans is the transformed measurement, M is the original measurement, b is the within-group slope regression of the log M versus log SL, SL is the standard length of the fish and SL mean is the overall mean of the standard length (correlation < 0.5 for all variables).

From the final analysis, Standard length (SL) was excluded, since SL was used as a basis for transformation [64, 65]. All statistical analyses were performed for combined sexes as there were no significant differences of tested variables between the sexes (*p*>0.05). One-way analysis of variance (ANOVA) was performed for each character between the species and significant variables were retained [66, 67]. Tukey’s-b significance difference test was executed as a post-hoc multiple-comparisons test. Subsequently, significant variables were subjected to PCA, DFA and CA. The holdout leave-one-out cross-validation procedures proposed by Lachenbruch [68], were also carried out to calculate misclassification rate of DFA. Average shape of all specimens of each species were computed and aligned using tpsRelw software to perform an analysis of relative warps (RW), i.e., a principal components analysis of shape variation relative to spatial scale [69–71]. Each spline is a visualization of the group mean relative to the grand mean. Statistical analyses were performed with the computer software programs SPSS 16.0 and PAST 1.47. A hierarchical cluster analysis based on UPGMA was carried out on Mahalanobis distances. Software STATISTICA was used to test the significance of Mahalanobis distance between species.

### Ethics-statement

Fish samples were obtained from the wild, directly from the commercial catches. Samples of all fish species were procured from local fish markets after commercial consignment with the fish vender. Sites from where fishes were collected fell outside Protected Areas (PAs) and therefore no permits were required from the State Forest and Wildlife Department. Fish were captured by gill nets. For morphometric and meristic study, fish, if alive were euthanized with MS222 (Sigma) to ameliorate suffering and transported to the laboratory on ice to avoid damage to its morphological characters that are crucial for taxonomic investigations. The Ethical committee of Lucknow University, Lucknow India has approved the design and implementation of the study.

## Results

### Traditional morphometric analysis

**Fig 6:**
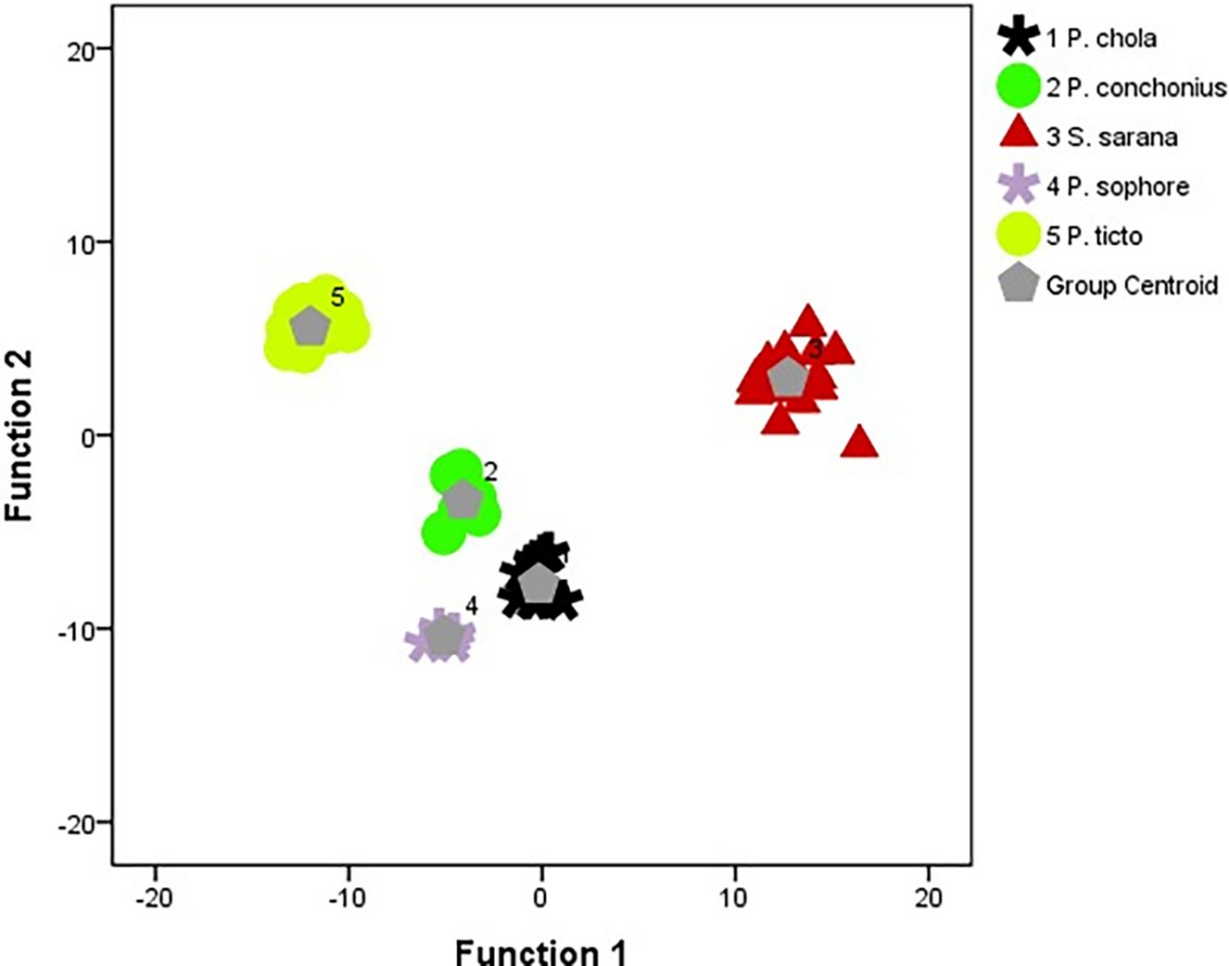
Discriminant function plot from traditional morphological variables. (Group Centroids: 1: *P*. *chola;* 2: *P*. *conchonius;* 3: *S*. *sarana;* 4: *P*. *sophore* 5: *P*. *ticto*).

In one way ANOVA, 29 characters were significant out of 32 characters (*p*<0.05). These were then subjected to principle component analysis (PCA). PCA extracted 8 principal components at Jolliffe’s rule with eigenvalues of at least 0.7 responsible for 85.30% variations in which PC1 accounts for maximum variation in the samples which is 39.78%, and PC2 contributes 18.65%. Significant variables were subjected to DFA and a resultant 100.0% of original grouped cases correctly classified. Also, 100.0% of cross-validated grouped cases were correctly categorized (Table 3). The DFA plot is shown in Fig 6.

**Table 3:** Discriminant function analysis on traditional characters among five fish species (100.0% of original grouped cases correctly classified and 100.0% of cross-validated grouped cases correctly classified).

Predicted Group Membership							Total
Original percentage (%)	Species	***P*. *chola***	***P*. *conchonious***	***S*. *sarana***	***P*. *sophore***	***P*. *ticto***	
	***P*. *chola***	100.0	.0	.0	.0	.0	100.0
	***P*. *conchonious***	.0	100.0	.0	.0	.0	100.0
	***S*. *sarana***	.0	.0	100.0	.0	.0	100.0
	***P*. *sophore***	.0	.0	.0	100.0	.0	100.0
	***P*. *ticto***	.0	.0	.0	.0	100.0	100.0
Cross validated percentage (%)	***P*. *chola***	100.0	.0	.0	.0	.0	100.0
	***P*. *conchonious***	.0	100.0	.0	.0	.0	100.0
	***S*. *sarana***	.0	.0	100.0	.0	.0	100.0
	***P*. *sophore***	.0	.0	.0	100.0	.0	100.0
	***P*. *ticto***	.0	.0	.0	.0	100.0	100.0

DFA extracted 8 discriminant variables (dorsal and anal fin length, head depth at the eye, number of pre-dorsal scales, number of caudal circumferential scales, vertebrae counts, dorsal and pectoral fin rays, S2 Table). Combination of these characters is responsible for variations among species. Mahalanobis distances from traditional morphometric data suggested that the five species are at significant distance from each other (Table 4).

**Table 4:** Pair wise matrix of Mahalanobis distances between the centroids of the clusters (above diagonal) and *p* value (below diagonal) of discriminant traditional morphological characters among five fish species.

	*P*. *chola*	*P*. *conchonius*	*S*. *sarana*	*P*. *sophore*	*P*. *ticto*
***P*. *chola***		185.2358	576.396	119.0583	688.970
***P*. *conchonius***	1.63593E-06*		850.503	278.1810	468.848
***S*. *sarana***	2.13803E-21*	1.39695E-18*		879.3172	1315.182
***P*. *sophore***	0.00563457*	0.371188	1.1027E-17*		745.952
***P*. *ticto***	1.03384E-15*	2.50182E-07*	2.86447E-33*	2.40004E-09*	

(*p <0.001)

**Fig 7:**
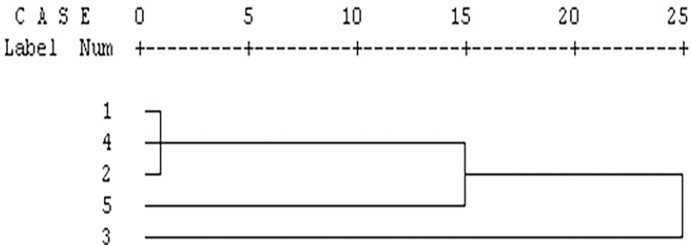
Dendrogram derived from UPGMA cluster analysis based on the Mahalanobis distance between the species centroids from traditional morphological variables. (1: *P*. *chola;* 2: *P*. *conchonius;* 3: *S*. *sarana;* 4: *P*. *sophore* 5: *P*. *ticto*).

On the basis of morphometric and meristic data, a dendrogram of the species was derived by the unweighted pair group (UPGMA) cluster analysis. The UPGMA cluster analysis based on the Mahalanobis distance between group centroids showed that the five species produced two major clusters. *P*. *chola*, *P*. *sophore* and *P*. *conchonius* belong to one cluster and *P*. *ticto* belongs to sub-branch of the same cluster while *S*. *sarana* is most distinctly placed (Fig 7).

### Landmark-based analysis

In one way ANOVA, all the 90 characters were found significant (*p*<0.05). Tukey’s-b post hoc test revealed that 13 characters grouped 5 supposed species into 5 groups. Although all the 90 characters grouped five species into more than single homogenous subsets therefore all the characters were retained for further analysis. These characters were subjected to PCA, at Jolliffe’s rule with eigenvalues of at least 0.7. In total, 4 principal components were extracted through PCA responsible for 96.45% variation. The first two components extracted, accounts for a total variance of 94.26%, in which the first principal component (PC1) accounts for 92.38% while second PC2 contributes 1.89%.

**Fig 8:**
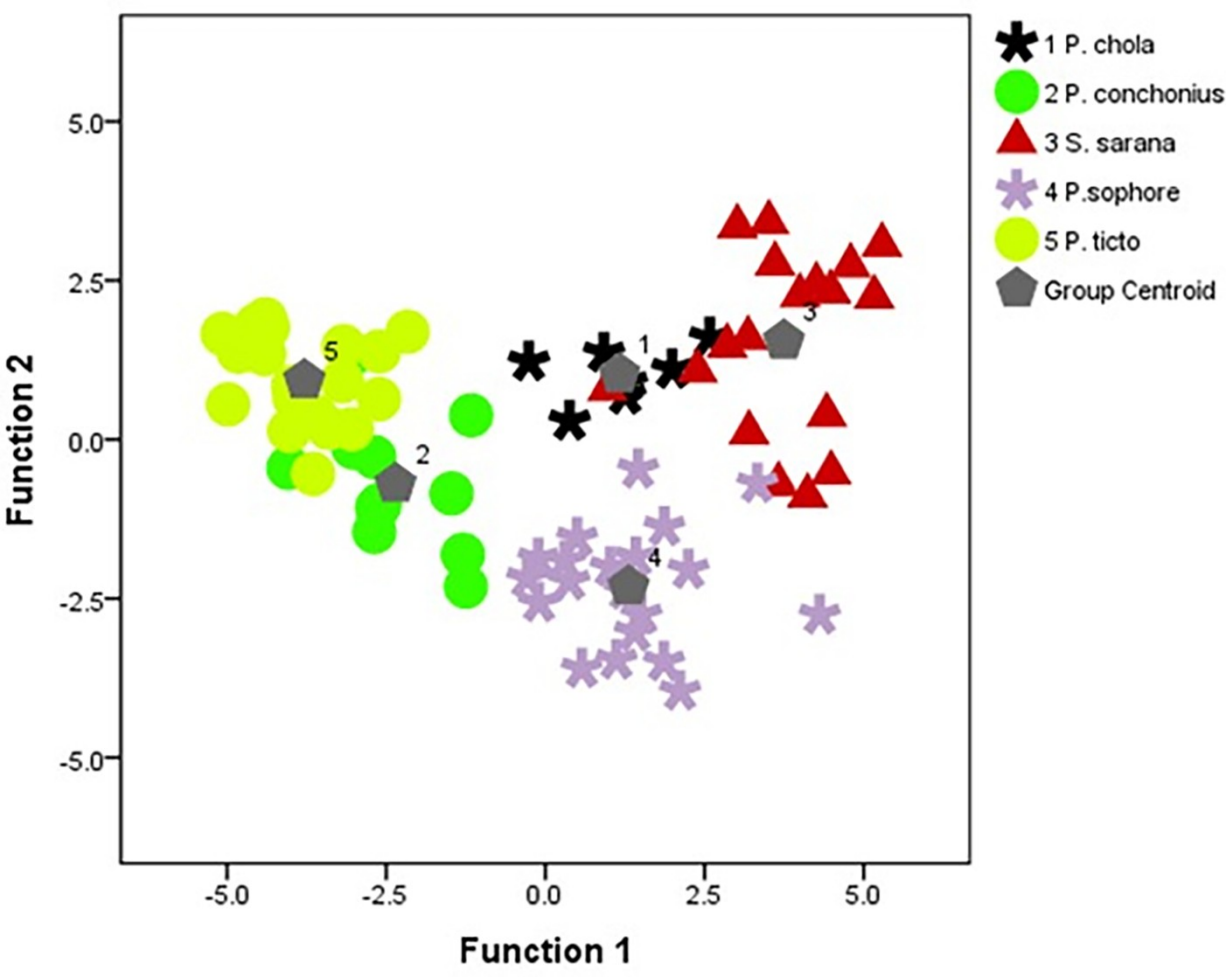
Discriminant function plot from truss morphometric variables. (Group Centroids: 1: *P*. *chola;* 2: *P*. *conchonius;* 3: *S*. *sarana;* 4: *P*. *sophore* 5: *P*. *ticto*).

In discriminant function analysis (DFA), 96.1% of original grouped cases were correctly classified and 92.1% of cross-validated grouped cases correctly classified (Table 5). Based on the discriminant function analysis, combined group plots of the five groups showing the differences among the groups and illustrated that there was little overlapping among the groups (Fig 8).

**Table 5:** Discriminant function analysis on truss characters among five fish species (96.1% of original grouped cases correctly classified and 92.1% of cross-validated grouped cases correctly classified).

Predicted Group Membership							Total
Original percentage (%)	Species	***P*. *chola***	***P*. *conchonious***	***S*. *sarana***	***P*. *sophore***	***P*. *ticto***	
	***P*. *chola***	100.0	.0	.0	.0	.0	100.0
	***P*. *conchonious***	.0	90.9	.0	.0	9.1	100.0
	***S*. *sarana***	5.6	.0	94.4	.0	.0	100.0
	***P*. *sophore***	.0	.0	5.0	95.0	.0	100.0
	***P*. *ticto***	.0	.0	.0	.0	100.0	100.0
Cross validated percentage (%)	***P*. *chola***	100.0	.0	.0	.0	.0	100.0
	***P*. *conchonious***	9.1	81.8	.0	.0	9.1	100.0
	***S*. *sarana***	11.1	.0	88.9	.0	.0	100.0
	***P*. *sophore***	.0	5.0	5.0	90.0	.0	100.0
	***P*. *ticto***	.0	.0	.0	.0	100.0	100.0

**Fig 9:**
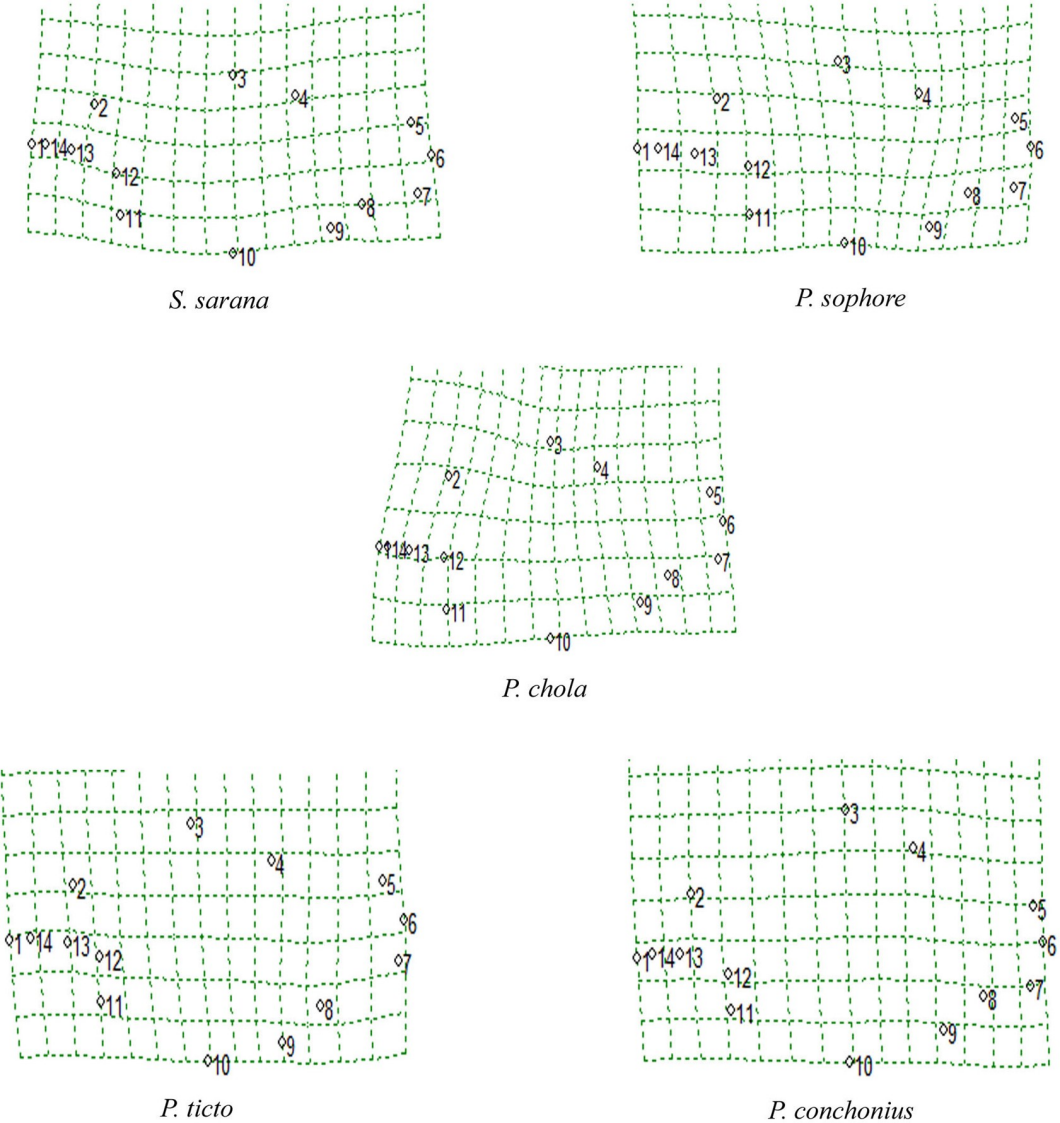
Landmark based geometric morphometric analysis showing the variation of the body shapes among species.

Eight discriminant variables were extracted (distance between origin of dorsal fin to end of dorsal fin and insertion of pelvic fin, distance between end of dorsal fin to origin of anal fin, distance between anterior attachment of ventral membrane from caudal fin to insertion of pectoral fin, head length, distance between origin of dorsal fin and anterior attachment of dorsal membrane from caudal fin, distance between origin of anal fin to posterior end of eye and distance between insertion of pelvic fin to insertion of pectoral fin), which were responsible for variation among species. Mahalanobis distances from truss morphometric data suggested that the five species are at a significant distance from each other (Table 6). Relative warps (RW) of each species’ shape variation were easier to interpret through localization of fourteen landmarks on the entire body form on diagram grids (Fig 9). Apparently, shape variations are captured due to the relative positions of the following set of landmarks 3, 4, 5 and 8.

**Table 6:** Pair wise matrix of Mahalanobis distances between the centroids of the clusters (above diagonal) and *p* value (below diagonal) of discriminant truss morphometric characters among five fish species.

	*P*. *chola*	*P*. *conchonius*	*S*. *sarana*	*P*. *sophore*	*P*. *ticto*
***P*. *chola***		3727.456	2135.690	3030.644	3332.055
***P*. *conchonius***	0.000974045*		6499.911	7519.836	817.258
***S*. *sarana***	0.00411839*	8.45015E-09*		750.174	4348.666
***P*. *sophore***	4.14166E-05*	4.70306E-05*	1.65E-07*		5038.670
***P*. *ticto***	5.74079E-08*	0.000295858*	2.4811E-15*	4.20184E-14*	

(*p <0.001)

**Fig 10:**
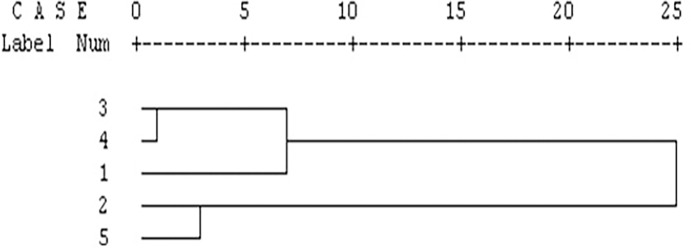
Dendrogram derived from UPGMA cluster analysis based on the Mahalanobis distance between the species centroids from truss variables. (1: *P*. *chola;* 2: *P*. *conchonius;* 3: *S*. *sarana;* 4: *P*. *sophore* 5: *P*. *ticto*).

A dendrogram of the species based on the landmark-distances data was derived by the unweighted pair group (UPGMA) cluster analysis. The UPGMA cluster analysis based on the Mahalanobis distance between group centroids showed that the five species on the basis of similarity in body shape produced two major clusters. *S*. *sarana* and *P*. *sophore* belong to the first cluster (cluster I) and *P*. *chola* placed as sub-branch while in another branch *P*. *conchonious* and *P*. *ticto* grouped together (cluster II) (Fig 10).

## Discussion

The morphometric variations based on traditional (body measurements and meristic characters) and truss network analysis of five species of Barbinae from Ganga river system of northern India using multivariate analysis (PCA, DFA, RW and CA) are found to be valid for discrimination among five species with varying in the degree of differentiation. The dissimilarity of the confirmation for divergence among species probably reflects our use of a different set of characters/landmarks in the traditional and truss methods, which incorporated a different set of measurements. Furthermore, all the analyses applied in the present study, have sufficient statistical capacity to discriminate among *P*. *sophore*, *P*. *chola*, *P*. *ticto*, *P*. *conchonius* and *S*. *sarana*. It is encouraging and suggests that previous morphometric studies using traditional measures can be reliable.

In the present study, the most significant measures taken into account for discrimination through traditional analysis were the length of the dorsal fin, length of the anal fin, head depth at the eye, number of pre-dorsal scales, number of caudal circumferential scales, dorsal fin rays, pectoral fin rays, and vertebrae count. Discriminating characters extracted through truss analysis were related to anterior, posterior, dorsal, lateral and ventral distances. Geometric morphometry based relative warps of average shape also provide evidence on the distinctness of species.

Meristic characters like pre-dorsal scales, caudal circumferential scales, vertebrae count, number of dorsal and pectoral fin rays were found to be helpful in discriminating these species. De Silva and Liyanage [72] opined on the basis of their study that meristic characters are more effective than morphometric characters for discriminating the *Puntius* genus. Kotalawala and Jinadasa [73] also reported that meristic characters (counts of lateral line scales, gill rakers, pectoral rays, and vertebrae) were helpful for differentiating species of *Puntius*. Other remaining meristic characters showed a moderate degree of overlap among selected species. Caudal fin rays were found to be a common and non-variable character in all the species. Dorsal fin rays counts are in agreement with Saroniya et al [32] though incongruent with the findings of Day [74], Srivastava [75], Hamilton [76], Datta Munshi and Srivastava [77], Talwar and Jhingran [56]. No changes were reported in meristic counts with the increase in fish body length. The similar observation was reported by Rajasekaran and Sivakumar [26], Saroniya et al [32], Vladykov [78], Talwar and Jhingran [79] and Muhammad Zafar et al [80]. Variation in vertebrae counts among the *Puntius* species were found to be one of the discriminating variables though, less precisely contributing in the differentiation of species, as this character was found overlapping among the species *P*. *ticto* (vertebrae count 25–28), *P*. *sophore* (26), *P*. *chola* (27–29), *P*. *conchonius* (25), but not *S*. *sarana* (32–34). Shantakumar and Vishwanath [27] reported similar trends in vertebrae counts of *Puntius* species in consideration. Though, Weitzman and Cobb [81], Jenkins and Lachner [82] opined that vertebrae counts could be employed in the discrimination of genera and species. A single pair maxillary barbels present in *P*. *chola*, a pair of maxillary and a pair of rostral barbels are present in *S*. *sarana* while other species lacking barbels. Notably, the presence of barbels in every individual of a species increases the systemic importance of this characteristic [82], usage of barbels at the generic level has been corroborated [83].

The results of the traditional morphometric study revealed that there were significant variations in the morphometric characters and also significant differences were detected in the meristic counts among selected species. These results confirmed that the differences among the species reflected the varied quantity of differences as depicted by truss analysis. Similar findings were observed in *Labeo* genus by Lal et al [53]. DFA highlighted that investigated species can be precisely differentiated, distinctly clustered with only a partial overlap among them with applied truss analysis, but interestingly in traditional analysis, no overlap was visible.

To show hierarchical similarity clusters were built from traditional and truss morphometric data, resultant cluster topologies were not similar. Cluster based on traditional analysis shows that *P*. *chola*, *P*. *sophore*, *P*. *conchonius* and *P*. *ticto* belongs to one major group and *S*. *sarana* in another group. Traditional-based results are in partial congruence with that of earlier reports based on morphometric characteristics and meristic counts [see 79, 84, 85, 86]. In the present study, cluster drawn through the truss analysis showing a close relationship between *P*. *chola* and *P*. *sophore* and also between *P*. *conchonius* and *P*. *ticto*. Truss-based results were broadly congruent with previously proposed hypotheses of species relationships based on molecular phylogenetic studies [10, 28, 87] By contrast, sequences amplified through CO1 showed *P*. *ticto* highly resembled to *P*. *sarana* [88].

Utilizing standard methods, morphometric trees can simply be compared to molecular phylogenies trees to come up with a conclusion. Not to mention there is disagreement to the use of morphometric data in systematic contexts [89], although both morphometric and phylogeny contribute to a common fascination in the examination of morphological variation [89, 90]. Separation of species mostly reveals evolutionary relatedness if variables from different morphometric characters are utilized in a particular analysis [91]. Morphological differences are supposed to be characterized by gaps among taxonomic group, therefore morphological data are significant in biological systematics. These gaps may occur as a result of a number of evolutionary processes [89, 92]. On the whole, morphometric data consist of more than one species with different morphometric characters probably have a phylogenetic element responsible for variation in shape.

Although, molecular genetics techniques have been frequently employed to identify the distinctness among species, the feasibility and importance of classical techniques cannot be denied. Further, morphometric methods have been found to be powerful and feasible for investigating taxonomic problems with advances in improved data collection, a better description of shape, and the potential of new analytical techniques. In different biological contexts, the geometric method, provides shape-related additional information present in the relative locations of landmarks [93]. Geometric morphometrics (GM) is used as a potent tool to analyze body form. Most of the software used in GM is freely available, user-friendly and offers an integrated approach that explains its utility as a better resource, such that Geometric-based RW method acquires enhanced discriminating power [94–97]. Subsequently, in the present study an effort was made to incorporate this. RW visualized differences in the body form are highly effective for discriminating selected *Puntius* species. Apparently, these differences are attributed mainly to the curvature of the body. However, results acquired were initial and analytic but able to supplement present study,

Fish show more noteworthy variation in morphometric attributes both intraspecific and between species when compared to other vertebrates and are more disposed to ecological changes [98, 99]. Mallet [100] describes species as identifiable ‘morphological and genotypic clusters’. Morphometric characters are developed from the combination of genotypic and environmental factors, and they are governed by natural selection [101]. Therefore to authenticate the morphometric differences and for an enhanced perceptive about these examined species, genetic-level studies can be performed.

In the present study, both analyses independently discriminated selected species into their groups. This indicated that the traditional system, as well as a truss, could be effectively used for morphological differentiation of these species. Geometric-morphometric-based relative warps provide additional information about the change in body form of these species. To summarize, findings of the present study suggest that truss technique is helpful in solving taxonomic ambiguity through quantifying shape variation. Questionable genera, here *Puntius*, could be differentiated, even with low sample sizes, provided they are used in combination with traditional morphological analysis. Thus, the present study offers the useful insight on the application and complementary role of truss analysis with traditional morphometrics.

## Supporting information

S1 Table Description of traditional morphometric measurements taken on the body of five selected species of *Puntius*. (DOCX)

S2 Table Standardized canonical discriminant function coefficients. Showing eight discriminative variables generated through traditional analysis.
(DOCX)

S1 File Raw dataset of the traditional morphometric analyses. (XLSX)

S2 File Raw dataset of the truss morphometric analyses. (XLSX)
