# Supplementary material for: Taxonomic validation of five fish species of subfamily Barbinae from the Ganga river system of northern India using traditional and truss analyses
Source: PLoS One. 2018 Oct 26;13(10):e0206031. doi: 10.1371/journal.pone.0206031 (PMC6203374; doi:10.1371/journal.pone.0206031)
Supplement: S1 Table — (DOCX) [file pone.0206031.s001.docx]

| **S.**  **No.** | **Code** | **Morphometric Characters** | **Definitions** |
| --- | --- | --- | --- |
| 1 | SL | Standard length | The straight-line measurement from anterior end of snout to base of caudal fin |
| 2 | MBD | Maximum body depth | The straight-line measurement between dorsal and ventral surfaces at the point of origin of dorsal fin |
| 3 | MBW | Maximum body width | The greatest straight-line measurement from one side to other; Widest transverse of the body |
| 4 | PDL | Pre-dorsal length | The straight-line measurement from anterior end of snout to origin of first dorsal fin |
| 5 | PVL | Pre-ventral length | The straight-line measurement from anterior end of snout to origin of the pelvic fin |
| 6 | PAL | Pre-anal Length | The straight-line measurement taken from anterior tip of snout to posterior edge of the anus |
| 7 | PANSL | Pre-anus length | The straight-line measurement taken from anterior tip of snout to anterior edge of the anus |
| 8 | LCPD | Length of caudal peduncle | The straight-line measurement taken from the base of the anal fin to the base of the caudal fin |
| 9 | DCPD | Depth of caudal peduncle | straight-line measurement at the greatest depth from dorsal to ventral surface of the caudal peduncle |
| 10 | LDF | Length of dorsal fin | The straight-line measurement from proximal to distal end of dorsal fin |
| 11 | LPF | Length of pectoral fin | The straight-line measurement from Proximal to distal end of pectoral fin |
| 12 | LVF | Length of ventral fin | Measurement from Proximal to distal end of pelvic fin |
| 13 | LAF | Length of anal fin | Measurement from Proximal to distal end of anal fin |
| 14 | PAL | Pelvic axial length | Measurement from distal end of pectoral fin to anal pore |
| 15 | HL | Head length | Measurement from anterior end of snout to posterior edge of operculum |
| 16 | ED | Eye diameter | The straight-line measurement from anterior to posterior end of the eye orbit |
| 17 | PROL | Pre-orbital length | The straight-line measurement taken from posterior end of the orbit to the posterior most end of the operculum |
| 18 | POOL | Post-orbital length | The measurement taken from posterior margin of eye orbit to the end of the |
| 19 | IOD | Inter-orbital distance | Measurement taken from the upper margin of right eye orbit to the upper margin of the left orbit as measured from dorsal surface |
| 20 | IND | Inter-narial distance | Measurement of least distance between two nostrils |
| 21 | MHW | Maximum head width | Widest transverse measurement of the head |
| 22 | HDE | Head depth at eye | Measurement taken between the dorsal and the ventral surfaces of the head, perpendicularly through the eye |
| 23 | HDN | Head Depth at Nape | Measurement taken between the dorsal and the ventral surfaces of the head, perpendicularly through the nape |
