# Supplementary material for: Taxonomic validation of five fish species of subfamily Barbinae from the Ganga river system of northern India using traditional and truss analyses
Source: PLoS One. 2018 Oct 26;13(10):e0206031. doi: 10.1371/journal.pone.0206031 (PMC6203374; doi:10.1371/journal.pone.0206031)
Supplement: S2 Table — Showing eight discriminative variables generated through traditional analysis. (DOCX) [file pone.0206031.s002.docx]

| **Characters** | **Function** | | | |
| --- | --- | --- | --- | --- |
|  | 1 | 2 | 3 | 4 |
| Length of Dorsal Fin | -.425 | .141 | -.267 | -.195 |
| Length of Anal Fin | .356 | .386 | .258 | -.328 |
| Head Depth at Eye | .447 | .318 | .203 | -.148 |
| Pre-dorsal Scales | .574 | .602 | -.654 | -.152 |
| Circumferential Scales | .462 | -.443 | .657 | -.042 |
| Dorsal Fin Rays | .101 | .508 | .292 | .818 |
| Pectoral Fin Rays | .292 | -.915 | -.137 | .484 |
| Vertebrae Counts | .739 | .048 | .414 | -.248 |
